# Biographical Sketch of the Late Sir Charles Bell

**Published:** 1842-08-13

**Authors:** 


***Biographical Sketch of the late Sir Charles Bell, K.H. F.R.S. &c.***—This eminent surgeon and physiologist, whose recent death has deprived the profession of one of its brightest ornaments was the youngest son of the Rev. William Bell, a clergyman of the Episcopal Church of Scotland.

He was born in the year 1775, and received his education in the High School of Edinburgh. He was still very young when he began the study of anatomy under the celebrated John Bell, who, twelve years senior to his brother Charles, was already in the highest estimation as a lecturer and a surgeon, and to whom he was apprenticed in 1792.

To the instruction he received under this enthusiastic and highly gifted teacher, may, in great measure, be attributed the success with which Mr. Bell cultivated and improved the sciences of anatomy and physiology; and the ardor with which he at this time pursued his studies, is shewn in the publication of the first volume of his "System of Dissections," when he was yet only in his twenty-second year. This, his earliest work, bears that stamp of originality which characterized every production of his after life. It presents the germs of numerous important doctrines which he employed himself in developing in his later publications. The engravings contained in it, taken from original drawings by himself, with some etchings by his own hand, prove how accomplished he was as an artist at that time; and were the first indications of a power of using his pencil, which was not only useful for illustrating his lectures and works, but was the source to him in private life of never-failing pleasure.

In the year 1799, Mr. Bell became a Fellow of the Royal College of Surgeons of Edinburgh, and was shortly afterwards appointed Surgeon to the Royal Infirmary. Here he soon acquired reputation as an operator, by his simple and dexterous manner of using the scalpel alone in the operation of lithotomy. A few years afterwards he determined to relinquish his prospects in Edinburgh, and seek success in London. To this step he was led by many circumstances, amongst the principal of which was, perhaps, the excited state of party feeling at that time existing in Edinburgh, and from the annoyances of which he could scarcely hope to escape, as his brother, whose powers of sarcasm and peculiar aptness for controversial writing made him an object of fear and dislike to his opponents, was at this time leader of one of the parties which divided the profession.

Mr. Bell came to London in 1806, and very soon gained for himself a high reputation as a lecturer on anatomy and surgery. Nothing can more decidedly prove how great were his qualifications for the career on which he had embarked than his success at such a time. Unsupported—dependent on his own exertions alone—he was enabled to enter into competition with Cline, Abernethy, Cooper, and others scarcely less known, and to come off with honour. Himself an ardent lover of his profession, he had a manner, scarcely to be surpassed, of inspiring in others the same admiration, and he could invest with an interest before unfelt the dryest details of anatomical demonstration.

It was only to be expected, that, with these powers, combined with an unwearying devotion to his professional pursuits, and to the progress of his pupils, he should have been enabled—associated with Mr. Wilson, and afterwards with Mr. John Shaw, in the school of Great Windmill Street—to raise the character of that school to the highest pitch. His lectures could not fail to interest and instruct even those who were unable to appreciate that boldness of conception and originality of mind which were displayed in them, and which afterwards led to so great results. It was in this school that he first explained his original and beautiful views on the Nervous System—those views which have gained to himself immortality, and reflected honour on his country.

Mr. Bell's fame as a surgeon must at this time have stood very high in London: for, in 1812, he was elected surgeon to the Middlesex Hospital, after the severest contest ever known in that institution. From this time, till he quitted London, he lost no opportunity of giving clinical instruction to the pupils. Many of these lectures were published in this journal: and our readers will not think that we exceed in our praise, if we represent them as among the best models of that style of teaching to be found in medical literature.

Previous to his election, he had received, after a nominal examination, the diploma of the College of Surgeons of London.

His zeal in the cultivation of professional knowledge had been before, and it was subsequently, instanced by the alacrity with which he availed himself of the opportunities offered for the study of gun-shot wounds by the battle of Corunna, and afterwards by that of Waterloo. After the latter action, he and his brother-in-law, Mr. John Shaw, were the first surgeons from England who arrived at Brussels; and Mr. Bell was unremittingly employed in his attentions to the wounded, in taking cases, and in making those sketches and drawings which have been justly regarded as amongst the finest specimens of water-colour painting. For three successive days and nights was he engaged in operating on and dressing the wounded, and he had no fewer than 300 cases under his own personal care. The results of his observations are appended to his "System of Operative Surgery."

His appointment to the Professorship of Anatomy and Surgery to the College of Surgeons, a few years later, gave him an opportunity of displaying, in the most decisive manner, the depth and clearness of his reasoning powers, the originality of his mind, and his singularly felicitous method of imparting knowledge.

He chose for his subject, among others, one which was always a favourite with him, "The evidence of design in the formation of our frames;" and he was daily listened to with attention and delight by crowds of the most eminent men of the day both in and out of the profession. That his style combined a substantial and effective power of reasoning which satisfied the understanding, with an easy and impressive manner that bound his listener's attention to him, may be illustrated by a saying imputed to the present venerable Bishop of Durham, then a frequent attendant upon his lectures—"Sir Charles Bell's discourses convey to me the impression not so much of a lecture, as of a man *thinking aloud*."

To the delivery of these lectures we owe the publication of his two papers on "Animal Mechanics" in the Library for the Diffusion of Useful Knowledge, in which is illustrated, in his most happy manner, the idea, that the type of most of those mechanical contrivances which the labour and ingenuity of man have been so long in perfecting, may be found by the inquirer in the construction and adaptation of parts in the animal economy. The remarkably extensive circulation of these papers proves how favourably they were received by the public.

Following out the same train of thought, he was led, when selected by the trustees of the Earl of Bridgewater to write one of the treatises in fulfilment of the wishes of that nobleman, to choose the subject of the "Hand;" his essay on which is too well known and appreciated to need comment.

He was afterwards associated with Lord Brougham in the publication of the illustrated edition of "Paley's Evidences of Natural Theology"—a beautiful work, to which both his pen and pencil contributed largely.

His reputation as a man of science, in consequence of these publications and his physiological discoveries, rose so high, that on the accession of William IV. he was amongst the number of those who received the honour of knighthood as an acknowledgment of their high scientific merit. In the meantime, he continued with undiminished success to deliver his lectures in Great Windmill Street, until the year 1828, when he was induced, on the formation of the London University, to accept the chairs of Physiology and Surgery in that school; and to him it was allotted to give the first lecture delivered within its walls.

He did not long remain attached to this institution, the dissensions which arose soon after its establishment causing him to relinquish his Professorship. His connection with the Middlesex Hospital, however, continued until his appointment to the Chair of Surgery in the University of Edinburgh, in 1836, induced him to leave London, and to return to the scene of his early labours and his early success; taking with him the regrets of all who knew him, and the respect and esteem of the whole profession.

The life of Sir Charles Bell was one of incessant and laborious study. This is sufficiently evidenced by his numerous works. Few, however, except his friends, know how unremitting was his application to his duties as a Teacher—with what care and attention each lecture, though only clinical, was studied, even to his last course.

Before concluding this short sketch of one who for nearly half a century devoted his high talents to the advancement of our profession, it may be expected that we should say a few words on his private deportment in social life. His manners, we would say, were characterized by unpretending simplicity, combined with an openness and cheerfulness of demeanor, the best signs of an honourable and self-respecting mind. Refined in his tastes—blending in his manner great courtesy, with a dignity which commanded respect—his conversation flowing easily and naturally from the sources of his richly-stored and fertile mind, aided by his retentive and ready memory, and heightened by a vein of true and refined wit—he was in every way calculated to be an ornament to society.

With his singleness of character were united great firmness and moral courage: the fear of consequences never deterred him from inflexibly following what he considered his duty; his energy of mind often drew him into a forgetfulness of every object foreign to that which was occupying him; of what the world would have considered his interests he was never careful. Hence he was often regarded by those who did not know him as cold, indifferent, and severe; persons who really knew him will never cease to remember how entirely the reverse was his true disposition. He had a warmth of feeling which made him ever keenly alive to the happiness or misfortunes of his friends; ever ready to afford them his advice and assistance, and to promote their welfare to the utmost of his power.

Constantly exercising, even in relaxation, his acute powers of observation and natural ingenuity, objects to occupy and interest him were seldom wanting. Indeed, he displayed, in a remarkable manner, an enlightened sympathy with every, even the meanest, work of nature. From this cause it may have arisen that his pleasures and solacements from the toils of business were usually obtained from simple sources. One principal feature of his character was his being an ardent admirer of the beauties of the country, to which no doubt his facility and admirable skill in sketching from nature greatly contributed. For many of his latter years he was a keen fisher, a sport the love of which was partly cherished on account of the healthful exercise it secured to him; but more, perhaps, for the delightful rural scenes into which it carried him. In fine, we may sum up his private character by saying, that, joined to his highly philosophical and powerful intellect, he possessed a heart warm and generous, and susceptible of the kindliest impressions. Gifted with qualities to command admiration in others, it was the affection of those immediately around him, and connected with him by long friendship, for which he seemed principally to live.—*London Med. Gazette*. June 3, 1842.

